# Practice of exclusive breastfeeding and its associated factors in a suburban area in Angola: a cross-sectional study

**DOI:** 10.1590/1516-3180.2018.0262161118

**Published:** 2018-12-13

**Authors:** Susana Valéria Dalcastagnê, Elsa Regina Justo Giugliani, Luciana Neves Nunes, Lisiane Hauser, Camila Giugliani

**Affiliations:** I MD, MSc. Physician, Community Health Service, Hospital Nossa Senhora da Conceição, Porto Alegre (RS), Brazil.; II MD, PhD. Physician, Assistant Professor, Department of Pediatrics, Universidade Federal do Rio Grande do Sul (UFRGS), Porto Alegre (RS), Brazil.; III PhD. Statistician and Associate Professor, Department of Statistics and Postgraduate Program on Epidemiology, Universidade Federal do Rio Grande do Sul (UFRGS), Porto Alegre (RS), Brazil.; IV PhD. Statistician and Statistical Consultant, Telessaude-RS Scientific Technical Nucleus; and Professor, São Francisco de Assis College, Porto Alegre (RS), Brazil.; V MD, PhD. Physician and Assistant Professor, Department of Social Medicine and Postgraduate Program on Epidemiology, Universidade Federal do Rio Grande do Sul (UFRGS), Porto Alegre (RS), Brazil.

**Keywords:** Breast feeding, Infant nutrition disorders, Angola

## Abstract

**BACKGROUND:**

Exclusive breastfeeding for six months is one of the measures with highest impact on prevention of child deaths. The determinants of breastfeeding practices are complex and differ between populations. This study aimed to identify factors associated with the prevalence of exclusive breastfeeding in a suburban area in Angola.

**DESIGN AND SETTING:**

Population-based cross-sectional study in the municipality of Cacuaco, Luanda.

**METHODS:**

A random sample of children under two years of age and their mothers was included. ­Prevalence ratios (PR) were estimated using Poisson regression based on a hierarchical model.

**RESULTS:**

749 children and their mothers were surveyed, including 274 children under six months. Theprevalence of exclusive breastfeeding among children under six months was 51.5% (95% confidence interval, CI, 46.3-56.6%). Four variables were positively associated with exclusive breastfeeding at ages of under six months: number of prenatal visits (PR 1.11 for each visit after the first one; 95% CI 1.04-1.18), maternal occupation (other occupations versus self-employed) (PR 1.54; 95% CI 1.05-2.26), younger child age (PR 0.77 for each month; 95% CI 0.71-0.84) and female child (PR 1.34; 95% CI 1.02-1.76).

**CONCLUSIONS:**

Our findings showed that the prevalence of exclusive breastfeeding at six months was satisfactory, according to international recommendations. Factors associated with exclusive breastfeeding practices that had never been surveyed before in Angola were identified through this study. These data are particularly relevant in the context of high infant mortality and may be useful in planning actions aimed at improving child health through promotion of exclusive breastfeeding, in Angola and other countries.

## INTRODUCTION

Breastfeeding has proven to be an effective practice for preventing child deaths.^1^^,^^2^ It has been estimated that scaling up breastfeeding to a near universal level could prevent 823,000 child deaths, along with 20,000maternal deaths from breast cancer annually.^1^ Breastfeeding also has a significant impact on reducing morbidity from infectious diseases, especially gastrointestinal and respiratory diseases. There is evidence showing that it has a protective effect against a variety of illnesses over the short and long terms, and showing that it promotes cognitive development.^1^^,^^3^

Over the last two decades, since the World Health Organization (WHO) issued its recommendation of exclusive breastfeeding (EBF) for the first six months of life, followed by introduction of complementary feeding but with continued breastfeeding until at least two years of age,^4^ there has been a trend toward increased prevalence and duration of breastfeeding worldwide. However, the distribution of this trend has differed between different locations. The prevalence of exclusive breastfeeding in low-income and middle-income countries has increased by an average of 0.5% per year (from 24.9% in 1993 to 35.7% in 2013), and more sharply among more economically advantaged women.^1^

Breastfeeding practices can be influenced by historical, demographic, socioeconomic, cultural and individual factors. The success of breastfeeding depends on understanding these variables and on interventions at different levels.^5^

In Angola, a country still suffering the consequences of a civil war that lasted 27 years, there are few data on breastfeeding. Despite showing signs of socioeconomic recovery, this country has an under-five mortality rate of 167.4 deaths per 1000 live births: the highest rate in the world.^6^^,^^7^ Data from a national survey conducted in 2001 (United Nations Children’s Fund [UNICEF] Multiple Indicator Cluster Survey [MICS]) showed that the prevalence of EBF was only 13.6% among children under four months of age.^8^ However, no studies involving surveys of the determinants of exclusive breastfeeding in this country have been conducted.

To fill this gap, the present study aimed to identify factors associated with the practice of exclusive breastfeeding among children under six months of age in a municipality in the metropolitan area of Luanda, Angola. In addition, we estimated the prevalences of exclusive breastfeeding among children under 6months, of breastfeeding among children under 24months, and of continued breastfeeding from 12 to 24months.

## OBJECTIVE

The aim of this study was to identify factors associated with the prevalence of exclusive breastfeeding in a suburban area in Angola.

## METHODS

This was a population-based cross-sectional study that was linked to a broader project entitled “Developing primary healthcare services in Angola: a proposal for assessment of the community health workers program,” in which data were collected from August 1 to September 26, 2010.

The study was conducted in Cacuaco (700,000 inhabitants at the time of data collection),^9^ which is a municipality in the metropolitan area of Luanda, the capital of Angola (total population of 25million).^10^This location was chosen because it was the first municipality to implement the community health workers program.

The eligible participants were all children aged 0 to 23months whose mothers lived in the survey area. If more than one child under two years of age lived in the same household, only the older child was included. In the case of twins, only the firstborn was included. Mothers who had lived in the survey area for less than one year or who did not live with their children were excluded.

Participants were considered lost to follow-up if their mothers were not found at home after at least three visits by interviewers to the household, on different days and at different times. They were considered to be refusals when their mothers refused to participate in the study.

For the original project, a sample of 700 children was calculated based on the estimated prevalence of some of the main outcomes under study (low body mass index-for-age and low height-for-age). Variation from 10% to 40% was assumed, at a precision level of 5% and considering a cluster design effect of 1.5. For the purposes of the present study, the prevalence of exclusive breastfeeding was assumed to depend on determinants that had previously been investigated in other countries in Africa, i.e. maternal education, family income, maternal occupation and child age.^11^^,^^12^^,^^13^ Using the same parameters (precision level of 5% and cluster design effect of 1.5), a sample size of 72 to 486 participants was found to be necessary (the large variation is due to the large number of determinants tested).

Participants were recruited from four districts, which were selected based on the following criteria: availability of neighborhood maps, authorization by residents’ committees and researcher safety. The districts were divided into microareas of approximately 100 households each. One household was randomly selected in each microarea as a starting point for the survey, and every third house to the right of the index house was then visited by the interviewers.

The Angolan interviewers underwent five days of intensive training, after which four teams were assembled, each consisting of a field coordinator, four interviewers and an area supervisor. A structured questionnaire was applied to the mother and additional data were obtained from pregnancy and child health cards. Standardized anthropometric measurements were obtained by properly trained field coordinators, using Tanita digital scales and custom-made wooden stadiometers. All questionnaires were coded, scanned and entered into a database using the Teleform software.

Exclusive breastfeeding was assessed among children under six months of age and breastfeeding among children under 24months of age. In addition, breastfeeding was assessed among children aged 12 to 15.9months (continued breastfeeding at 12months) and among children aged 20 to 24months (continued breastfeeding at 24months). The information on breastfeeding that was obtained referred to the child’s feeding habits on the day of the interview (current status). The indicators were calculated in accordance with the WHO references,^14^ except for the prevalence of breastfeeding among children under 24months of age, for which we thought it would be useful to describe the total prevalence in the sample.

**Figure 1.**
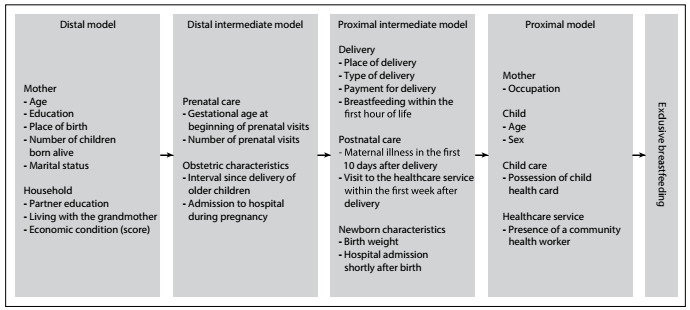
Hierarchical model of the determinants of exclusive breastfeeding.

The independent variables investigated are shown in Figure 1.

Economic status was assessed indirectly by means of a score that was obtained based on a previous study conducted in Ghana.^15^ Through this score, the participating families were stratified into different economic levels. The score was used in this study as a continuous variable, ranging from 0 to 10.

Quantitative variables were expressed as medians and quartiles (Q1 and Q3), and categorical variables were expressed as frequencies and proportions with 95% confidence intervals (95% CI). Thefrequencies of the independent variables were also examined in the group of children under six months of age, stratified according to the rate of occurrence of exclusive breastfeeding.

Poisson regression with robust variance was used to estimate prevalence ratios (PR) for exclusive breastfeeding as an outcome, with the respective 95% CI. Variables were included in a multivariable model based on a hierarchical model (Figure 1) in which the exposure variables were classified into levels considering their proximity to the dependent variable, according to the conceptual basis for possible interrelationships involving the factors under study.^16^^,^^17^At each step of the analysis, the variables were adjusted for others at the same level of the hierarchical model and for those showing statistically significant associations at a P-value of up to 0.20 at previous levels. In the final model, P < 0.05 was considered significant. Statistical analyses were performed using the Statistical Package for the Social Sciences (SPSS), version 18.0, and using Stata 9.

The present study was approved by the Research Ethics Committee of the Federal University of Rio Grande do Sul (Universidade Federal do Rio Grande do Sul, UFRGS), under protocol no. 1025941, dated April 16, 2015. In this study, we analyzed secondary data from the database of the original project, which had been approved by the Research Ethics Committee of UFRGS under protocol no. 2008045, dated May 8, 2009. Data collection was authorized by the Health Department of Luanda Province, Angola. All participating mothers provided written informed consent to be interviewed for the study.

## RESULTS

**Figure 2.**
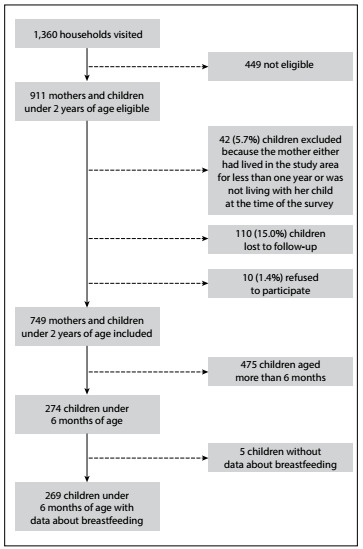
Flow diagram of study population.

A total of 1,360 households were visited, in 49microareas of four selected districts. Among the 911 eligible children in these households, 162 were excluded; 42 (5.7%) for the reasons described in the flowchart in Figure 2, 110 (15.0%) were lost to follow-up and in 10 cases (1.4%) the mothers refused to participate. Thefinal sample consisted of 749 children and their mothers. Table1shows the characteristics of the total sample (n = 749). Table 2 describes the features of the children under six months of age according to whether exclusive breastfeeding was practiced (n=269). Figure2 shows the flow of study subjects.

**Table 1.** Characteristics of the total sample in the municipality of Cacuaco, Angola, 2010 (n = 749)

Variable	Median or n (%)	Q1-Q3 or 95% confidence interval
(n when continuous variable)		
Maternal age (n = 744)	25	21-30
Maternal education (years of schooling) (n = 657)	6	4-8
Maternal place of birth		
Born in Luanda	76 (10.2)	(8.1-12.6)
Born in another province	669 (89.8)	(87.4-91.9)
Number of children born alive from the mother (n = 749)	3	2-5
Marital status		
Living with a partner	639 (85.3)	(82.6-87.7)
Not living with a partner	110 (14.7)	(12.2-17.4)
Partner education (years of schooling) (n = 490)	9	7-11
Living with the grandmother		
Yes	122 (16.3)	(13.7-19.1)
No	627 (83.7)	(80.9-86.3)
Economic condition (score) (n = 749)	7	5-7
Gestational age at beginning of prenatal visits*		
20 or more weeks	155 (46.1)	(40.7-51.6)
Less than 20 weeks	181 (53.9)	(48.4-59.3)
Number of prenatal visits (n = 369)*	4	3-5
Interval since delivery of older children (months) (n = 572)	33	(24.0-46.8)
Admission to hospital during pregnancy		
Yes	34 (4.6)	(3.2-6.3)
No	713 (95.4)	(93.7-96.8)
Place of delivery		
Home	228 (30.5)	(27.2-33.9)
Healthcare service	520 (69.5)	(66.1-72.8)
Type of delivery		
Cesarean	38 (5.1)	(3.6-6.9)
Vaginal	711 (94.9)	(93.1-96.4)
Payment for delivery		
No or did not recall	478 (64.1)	(60.5-67.5)
Yes	268 (35.9)	(32.5-39.5)
Maternal illness in the first 10 days after delivery		
Yes	141 (18.9)	(16.6-21.9)
No or did not recall	605 (81.1)	(78.1-83.9)
Breastfeeding within the first hour of life		
No	176 (23.5)	(20.5-26.7)
Yes	573 (76.5)	(73.3-79.5)
Visit to the healthcare service within the first week after delivery		
No or did not recall	453 (60.5)	(56.9-64.0)
Yes	296 (39.5)	(36.0-43.1)
Birth weight (kg) (n = 457)*	3.3	3.0-3.6
Child hospital admission shortly after birth		
Yes	18 (2.5)	(1.5-38.9)
No	715 (97.5)	(96.2-98.5)
Maternal occupation		
Self-employed	327 (43.9)	(40.3-47.5)
Housewife	316 (42.4)	(38.8-46.0)
Other	102 (13.7)	(11.3-16.3)
Child age (months) (n = 748)	10.1	4.2-16.0
Child age group (months)		
0 < 1	43 (5.7)	(4.2-7.7)
1 < 2	39 (5.2)	(3.7-7.1)
2 < 3	46 (6.2)	(4.5-8.1)
3 < 4	52 (7.0)	(5.2-9.0)
4 < 5	50 (6.7)	(5.0-8.70)
5 < 6	44 (5.9)	(4.3-7.8)
6 < 12	151 (20.2)	(17.4-23.3)
12 < 24	323 (43.2)	(39.6-46.8)
Child sex		
Male	361 (49.2)	(45.5-52.9)
Female	373 (50.8)	(47.1-54.5)
Possession of child health card		
No	103 (13.9)	(11.5-16.6)
Yes	639 (86.1)	(83.4-88.5)
Presence of a community health worker		
No	378 (50.7)	(46.7-54.1)
Yes	368 (49.3)	(45.5-52.8)

n = number of subjects; CI = confidence interval; Q1 = first quartile; Q3 = third quartile. *Variables with reduced n, because these data were collected from the child’s or pregnant woman’s health card.

**Table 2.** Characteristics of children under six months according to use of the practice of exclusive breastfeeding (n = 269)

Variable	Exclusive breastfeeding at under six months of age			
	Yes		No	
	n (%) or median	CI or Q1-Q3	n (%) or median	CI or Q1-Q3
Maternal age (n = 266)	26.5	22.0-30.0	24.0	19.3-30.0
Maternal education (years of schooling) (n = 232)	6.0	4.0-8.0	6.0	4.0-7.5
Maternal place of birth				
Born in Luanda province	17 (12.1)	(7.18-18.60)	17 (13.3)	(7.93-20.41)
Born in another province	124 (87.9)	(81.40-92.82)	111 (86.7)	(79.59-92.07)
Number of children born alive from the mother (n = 269)	3.0	2.0-5.0	3.0	1.0-5.0
Marital status				
Living with a partner	15 (10.6)	(6.08-16.94)	22 (17.2)	(11.10-24.86)
Not living with a partner	126 (89.4)	(83.06-93.92)	106 (82.8)	(75.14-88.90)
Partner education (years of schooling) (n = 172)	8.0	7.0-12.0	8.0	6.8-10.0
Living with the grandmother				
Yes	18 (12.8)	(7.74-19.42)	20 (15.6)	(9.81-23.09)
No	123 (87.2)	(80.58-92.26)	108 (84.4)	(76.91-90.19)
Economic condition (score) (n = 269)	7.0	5.0-7.0	5.0	5.0-7.0
Gestational age at beginning of prenatal visits				
20 or more week	41 (43.2)	(33.03-53.72)	42 (56)	(44.06-67.45)
Less than 20 weeks	54(56.8)	(46.28-66.97)	33 (44)	(32.55-55.94)
Number of prenatal visits (n = 186)	4.0	3.0-6.0	3.0	2.0-5.0
Interval since delivery of older children (months) (n = 206)	36.0	26.3-47.8	34.0	24.0-43.3
Admission to the hospital during pregnancy				
Yes	3 (2.1)	(4.40-6.09)	8 (6.3)	(2.74-11.94)
No	138 (97.9)	(93.91-99.56)	120 (93.7)	(88.06-97.26)
Place of delivery				
Home	37 (26.2)	(19.20-34.31)	41 (32.0)	(24.06-40.85)
Healthcare service	104 (73.8)	(65.69-80.80)	87 (68.0)	(59.15-75.94)
Type of delivery				
Cesarean	8 (5.7)	(2.46-10.80)	4 (3.1)	(0.86-7.81)
Vaginal	133 (94.3)	(89.13-97.52)	124 (96.9)	(92.19-99.14)
Payment for delivery				
No or did not recall	90 (64.3)	(55.75-72.20)	81 (63.8)	(54.78-72.12)
Yes	50 (35.7)	(27.80-44.25)	46 (36.2)	(27.88-45.22)
Maternal illness in the first 10 days after delivery				
Yes	29 (20.9)	(14.44-28.57)	26 (20.3)	(13.72-28.33)
No or did not recall	110 (79.1)	(71.43-85.56)	102 (79.7)	(71.67-86.28)
Breastfeeding within the first hour of life				
No	33 (23.4)	(16.69-31.27)	33 (25.8)	(18.46-34.26)
Yes	108 (76.6)	6(8.73-83.31)	95 (74.2)	(65.74-81.54)
Visit to the healthcare service within the first week after delivery				
No or did not recall	78 (55.3)	(46.72-63.69)	91 (71.1)	(62.42-78.76)
Yes	63 (44.7)	(36.31-53.28)	37 (28.9)	(21.24-37.58)
Birth weight (kg) (n = 186)	3.3	3.0-3.6	3.3	2.9-3.6
Child hospital admission shortly after birth				
Yes	3 (2.2)	(0.45-6.27)	1 (0.8)	(0.02-4.38)
No	134 (97.8)	(93.73-99.55)	125 (99.2)	(95.66-99.98)
Maternal occupation				
Self-employed	47 (33.3)	(25.63-41.76)	52 (41.3)	(32.58-50.38)
Housewife	68 (48.2)	(39.74-56.79)	62 (49.2)	(40.19-58.26)
Other	26 (18.5)	(12.41-25.84)	12 (9.5)	(5.02-16.05)
Child age (months) (n = 186)	2.1	1.0-3.5	4.2	2.8-5.1
Child sex				
Male	53 (41.1)	(32.50-50.09)	68 (53.1)	(44.11-62.00)
Female	76 (58.9)	(49.91-67.50)	60 (46.9)	(38.00-55.89)
Possession of child health card				
No	17 (12.1)	(7.18-18.60)	5 (3.9)	(12.80-8.88)
Yes	124 (87.9)	(81.40-92.82)	123 (96.1)	(91.12-98.72)
Presence of a community health worker				
No	70 (49.7)	(41.12-58.18)	66 (52.0)	(42.93-60.91)
Yes	71 (50.3)	(41.82-58.88)	61 (48.0)	(39.09-57.07)

n = number of subjects; CI = confidence interval; Q1 = first quartile; Q3 = third quartile.

Out of the total of 274 children under six months of age, 141 were being exclusively breastfed at the time of the interview. Thus,the prevalence of exclusive breastfeeding among children under six months was 51.5% (95% CI 46.3-56.6%). Out of the 749 children in the total sample, 638 were breastfed, regardless of exclusivity, thus resulting in a prevalence of breastfeeding among children under 24months of age of 85.2% (95% CI 82.4-87.7%). The prevalences of continued breastfeeding at 12 and 24months were 88.5% (95% CI 81.7-93.4%) and 45.8% (95% CI 36.7-55.2%), respectively.

Table 3 shows the prevalence ratio for the practice of exclusive breastfeeding among children under six months of age adjusted for predictors, based on the hierarchical model. At the proximal level, the following variables were positively associated with the practice of exclusive breastfeeding: number of prenatal visits, maternal occupation (not being a housewife or self-employed), younger child age and female child.

**Table 3.** Multivariable analysis using a hierarchical model for exclusive breastfeeding at under six months of age (n = 269)

Variables	Distal level		Intermediate distal level		Intermediate proximal level		Proximal level	
	PR (95% CI)	P-value	PR (95% CI)	P-value	PR (95% CI)	P-value	PR (95% CI)	P-value
Maternal age (n = 266)	1.00 (0.95-1.05)	0.867	-	-	-	-	-	-
Maternal education (years of schooling) (n = 232)	1.03 (0.96-1.11)	0.367	-	-	-	-	-	-
Maternal place of birth								
Born in Luanda	1.00	0.400						
Born in another province	1.22 (0.77-1.95)		-	-	-	-	-	-
Number of children born alive from the mother (n = 269)	1.03 (0.90-1.18)	0.670	-	-	-	-	-	-
Marital status								
Living with a partner	1.00	0.432						
Not living with a partner	0.76 (0.38-1.52)		-	-	-	-	-	-
Partner education (years of schooling) (n = 172)	1.04 (0.97-1.19)	0.269	-	-	-	-	-	-
Living with the grandmother								
Yes	1.00	0.474						
No	1.24 (0.69-2.24)		-	-	-	-	-	-
Economic condition (score) (n = 269)	1.02 (0.93-1.12)	0.653	-	-	-	-	-	-
Gestational age at beginning of prenatal visits								
20 or more weeks			1.00					
Less than 20 weeks	-	-	1.18 (0.85-1.62)	0.325	-	-	-	-
Number of prenatal visits (n = 186)	-	-	1.07 (0.98-1.17)	0.152	1.11 (1.00-1.22)	0.040	1.11 (1.04-1.18)	0.002
Interval since delivery of older children (months) (n = 206)	-	-	1.00 (1.00-1.01)	0.431	-	-	-	-
Admission to hospital during pregnancy								
Yes			1.00	0.499				
No	-	-	1.43 (0.51-4.04)		-	-	-	-
Place of delivery								
Home					1.00	0.938		
Healthcare service	-	-	-	-	0.98 (0.62-1.57)		-	-
Type of delivery								
Cesarean					1.00	0.252		
Vaginal	-	-	-	-	1.36 (0.62-1.56)		-	-
Payment for delivery								
No or did not recall					1.00	0.243		
Yes	-	-	-	-	1.22 (0.88-1.69)		-	-
Maternal illness in the first 10 days after delivery								
Yes					1.00	0.565		
No or did not recall	-	-	-	-	1.14 (0.73-1.79)		-	-
Breastfeeding within the first hour of life								
No					1.00	0.344		
Yes	-	-	-	-	0.84 (0.58-1.21)		-	-
Visit to the healthcare service within the first week after delivery								
No or did not recall					1.00	0.372		
Yes	-	-	-	-	1.15 (0.84-1.57)		-	-
Birth weight (kg) (n = 186)	-	-	-	-	0.94 (0.67-1.33)	0.741	-	-
Child hospital admission shortly after birth					
Yes					1.00	0.640		
No	-	-	-	-	0.81 (0.32-2.00)		-	-
Maternal occupation								
Self-employed							1.00	0.097
Housewife	-	-	-	-	-	-	1.28 (0.96-1.70)	
Other	-	-	-	-	-	-	1.54 (1.05-2.26)	0.027
Child age (months) (n = 186)	-	-	-	-	-	-	0.77 (0.71-0.84)	< 0.001
Child sex								
Male							1.00	0.033
Female	-	-	-	-	-	-	1.34 (1.02-1.76)	
Possession of child health card								
Yes							1.00	0.491
No	-	-	-	-	-	-	1.13 (0.80-1.58)	
Presence of a community health worker								
No							1.00	0.499
Yes	-	-	-	-	-	-	1.09 (0.85-1.39)	

PR = prevalence ratio; CI = confidence interval.

## DISCUSSION

The prevalence of exclusive breastfeeding among children under six months of age in our study population was 51.5%, which is considered a satisfactory rate according to the criteria adopted by WHO (50 to 89%).^18^ This rate was considerably higher than the exclusive breastfeeding rate of only 13.6% among children under 4months of age that was identified in 2001, in a national survey using data collected from 18 provinces in Angola.^8^ It was also higher than the overall world prevalence (38%) and the mean prevalences that have been found in countries in the East Asia and Pacific region (30%), Latin America and Caribbean (32%), South Asia (47%), Sub-Saharan Africa (36%) and Central Africa (25%), the region in which Angola is located.^6^

In Africa, there is considerable heterogeneity in the prevalence of exclusive breastfeeding among countries. Based on the present results, in our study population, the prevalence of exclusive breastfeeding among children under 6months of age was higher than that reported for Nigeria (17%), Congo (21%), Kenya (32%) and Mozambique (43%); comparable to that reported for Ethiopia (52%); and lower than that reported for Togo (62%), Uganda (63%) and Rwanda (85%).^6^ The prevalence found in our setting may reflect post-war improvements in social and economic conditions as well as in healthcare services, especially within the scope of primary healthcare. The municipality where the present study was conducted was the first to implement the community health workers (CHW) program as part of the process of revitalizing municipal healthcare services in Angola. This program was developed in 2006 with the purpose of reducing maternal and child morbidity and mortality.^19^ Investment in primary healthcare and improved access to health services are believed to have contributed towards dissemination of information on the importance of exclusive breastfeeding and on the harm done through early introduction of complementary foods, such as liquids, which is a common and culturally accepted practice.

The prevalence of continued breastfeeding at 24months is also heterogeneous among African countries: 82% in Ethiopia, 54% in Kenya, 50% in Nigeria, 37% in Ghana, 31% in South Africa and only 17% in Congo.^6^ Maintenance of breastfeeding for longer periods in Luanda may be explained, at least in part, by cultural issues and the positive effect of recent investments in healthcare. It is worth noting that, in Angola and other countries with high poverty rates, low household purchasing power makes it costly or even unfeasible for families to purchase other types of milk, which may contribute towards the high prevalence of breastfeeding.

In the present study, each prenatal visit after the first one was associated with an increase of 11% in the prevalence of exclusive breastfeeding among children under 6months of age. There is evidence that interventions during pregnancy aimed at promoting breastfeeding have a positive impact on its prevalence, especially among primiparous women.^20^ In a study conducted in Nigeria using data from a population survey involving more than 7,000mothers, attending four or more prenatal visits was also significantly associated with higher prevalence of exclusive breastfeeding (OR 2.7; 95% CI 1.04-7.01).^12^ Wishing to breastfeed, which is a strong determinant of successful breastfeeding, is a feeling generally developed during pregnancy, especially in the third trimester. This may therefore be influenced by information received during pregnancy. It has also been demonstrated that interventions within healthcare services, including prenatal counseling, are effective in promoting breastfeeding.^5^ The finding of the present study is important because it supports the number of prenatal visits as a predictor that, in addition to being an indicator of quality of care, has a direct impact on exclusive breastfeeding rates.

In families with children under six months of age whose mothers had formal employment (mostly working in the public or private sector), the prevalence of exclusive breastfeeding was observed to be 54% higher than among mothers who were ­self-employed. Thereis evidence that maternal employment may negatively affect breastfeeding, especially when the mother returns to work.^21^ However,work-related interventions to promote breastfeeding (such as maternity leave policies and support for breastfeeding at the workplace) appear to have a positive impact on breastfeeding indicators.^5^ In the present study, there was no difference between housewives and self-employed workers (the latter category was defined as a reference for comparison).

Conversely, having formal employment was a protective factor, compared with being self-employed. While mothers with formal employment are protected by law such that their income is guaranteed in the first months after birth^23^ and thus are more available to provide breastfeeding, mothers who are ­self-employed and have no right to social security benefits tend to return to work earlier, especially in scenarios of high socioeconomic vulnerability. Thus, these latter mothers, with or without their babies, are prematurely exposed to situations of insecurity and precariousness, which can negatively affect the practice of exclusive breastfeeding. Moreover, mothers with formal employment tend to have a more structured daily life and a more organized routine, including a support network that provides help with household chores.

Within the category “other occupations”, there was a significant number of students. The higher prevalence of exclusive breastfeeding in this group may also have been due to a stronger support network and the possibility of being absent from classes during the first months of the infant’s life, which thus would make these mothers more available to provide breastfeeding.

There was an association between older child age and lower prevalence of exclusive breastfeeding, with an estimated reduction of 33% for each extra month of life. This was an expected association that had already been widely reported.^11^^,^^12^^,^^13^^,^^17^^,^^24^ Thischild age-related decrease in the prevalence of exclusive breastfeeding may be influenced by unawareness of the optimal duration of exclusive breastfeeding and by the belief that breast milk alone is not sufficient for child nutrition as the infant approaches six months of life.

An association between the sex of the child and the practice of exclusive breastfeeding was also observed, with a 34% higher prevalence of exclusive breastfeeding among female children under six months of age. In a study conducted in Nigeria, female infants were twice as likely to be exclusively breastfed as were male infants (odds ratio, OR, 2.13; 95% CI, 1.03-4.39).^12^ This was also found among children surveyed in all Brazilian state capital cities.^26^ Althoughcommonly observed, there is no consensus regarding the explanation for this result.^17^ Some cultural factors, such as the belief that boys have a more voracious appetite and need higher energy intake than girls, which would be met through earlier introduction of complementary foods, may help explain this phenomenon. However,further studies are required in order to elucidate this association.

Higher maternal education levels are often associated with improved breastfeeding indicators.^17^^,^^27^^,^^28^ In our sample, although the number of years of education was not very low (half of the women had had more than six years of education), the overall low quality of education offered in Angola may not have allowed any differences in education level among the mothers to be impactful enough to change maternal behavior in relation to breastfeeding.

Family income is another variable that is often identified as a determinant of breastfeeding practices.^11^^,^^12^^,^^27^^,^^28^ According to Victora etal., the influence of this variable differs from one country to another, depending on the economic context of the region under study.^1^ In the present study, no association was found between family income and the prevalence of exclusive breastfeeding. Webelieve that the considerable homogeneity of the economic status of the families surveyed in our study might explain this result.

There is evidence showing that home visits by CHWs have a positive impact on maternal and child health outcomes, including breastfeeding practices.^29^^,^^30^ However, this was not confirmed in the present study. This may be related to the fact that, in the area surveyed, CHWs were working in an incipient manner that was relatively unstructured.

The present study had some limitations. Among these, we can highlight that the small number of events relating to certain independent variables may have negatively affected the statistical analysis (e.g. only three cases of twins and 38 cases of cesarean section). Therefore, the variable “twin pregnancy”, which was initially included in the analysis, had to be excluded from the final model. Also, the sample may have been too small to provide significant differences in some variables.

It was not possible to determine the cause-effect relationship between the variables in the associations that were found in the present study because of the study design. However, the results suggest that fewer prenatal visits are associated with lower likelihood that women will be able to care for their own health and their child’s health, and consequently lower likelihood of breastfeeding. The same applies to early return to informal work, as in the case of the mothers who were self-employed. Thus, our findings highlight the importance of providing high-quality prenatal care and adopting a broader view of breastfeeding. This implies taking into account women’s biopsychosocial context, in order to increase adherence to prenatal care and healthcare guidelines, especially those relating to breastfeeding.

## CONCLUSION

Based on the present results, the study population of Luanda had satisfactory indicators for exclusive breastfeeding practices, although lagging behind international targets. The results point towards the importance of high-quality prenatal care for exclusive breastfeeding promotion. Prenatal visits and other occasions of contact that pregnant women and mothers have with healthcare services should also be used to share knowledge and practices regarding child nutrition in an attempt to delay the introduction of complementary foods during the first six months of life, especially in relation to male infants. Moreover, the protective factors of formal employment and provision of social security benefits regarding maintenance of exclusive breastfeeding were also noteworthy. These findings may contribute towards current knowledge through indicating paths to be followed in order to improve the health of women and children, in Angola and other low-income countries.
